# Post-stroke insomnia in community-dwelling patients with chronic motor stroke: Physiological evidence and implications for stroke care

**DOI:** 10.1038/s41598-018-26630-y

**Published:** 2018-05-30

**Authors:** A. Sterr, M. Kuhn, C. Nissen, D. Ettine, S. Funk, B. Feige, R. Umarova, H. Urbach, C. Weiller, D. Riemann

**Affiliations:** 1 School of Psychology, University of Surrey, Guildford, UK; 2 Department of Psychiatry and Psychotherapy, Medical Center - University of Freiburg, Faculty of Medicine, University of Freiburg, Freiburg, Germany; 3 University Hospital of Psychiatry and Psychotherapy, Bern, Switzerland; 4 Sleep Wake Epilepsy Center, Neuroscience Center, Bern, Switzerland; 5 Department of Neurology, Medical Center, University of Freiburg, Freiburg, Germany; 6 Department of Neuroradiology, Medical Center - University of Freiburg, Faculty of Medicine, University of Freiburg, Freiburg, Germany; 7 Department of Neurology, University Hospital Bern, Bern, Switzerland

## Abstract

Questionnaire studies suggest that stroke patients experience sustained problems with sleep and daytime sleepiness, but physiological sleep studies focussing specifically on the chronic phase of stroke are lacking. Here we report for the first time physiological data of sleep and daytime sleepiness obtained through the two gold-standard methods, nocturnal polysomnography and the Multiple Sleep Latency Test. Data from community-dwelling patients with chronic right-hemispheric stroke (>12 months) were compared to sex- and age-matched controls. Behavioural and physiological measures suggested that stroke patients had poorer sleep with longer sleep latencies and lower sleep efficiency. Patients further spent more time awake during the night, and showed greater high-frequency power during nonREM sleep than controls. At the same time the Multiple Sleep Latency Test revealed greater wake efficiency in patients than controls. Importantly these findings were not due to group differences in sleep disordered breathing or periodic limb movements. Post-stroke insomnia is presently not adequately addressed within the care pathway for stroke. A holistic approach to rehabilitation and care provision, that includes targeted sleep interventions, is likely to enhance long-term outcome and quality of live in those living with chronic deficits after stroke.

## Introduction

A substantive body of evidence has documented a close relationship between stroke and sleep disordered breathing^1^. However other changes in sleep after stroke are less well researched^2^. According to a recent meta-analysis^3^ examining the literature on sleep physiology after stroke, the majority of these studies are conducted in inpatient populations, often lack an adequate control group and frequently do not include an adaptation night. The polysomnographic, (PSG)-derived evidence on sleep in stroke is therefore limited.

Physiological (polysomnographic, PSG) sleep studies are critical as this methodology affords the comprehensive and objective characterization of sleep parameters as well as the diagnosis of organic sleep disorders (including sleep apnea). Moreover, the theoretical conceptualization of the processes involved in sleep, and its relation to physical and mental health, has been largely derived from PSG studies in neurologically healthy volunteers. Physiological sleep studies in patients therefore represent a necessary step to draw on the wealth of knowledge obtained from basic sleep research. In addition, it is clear that sleep difficulties are a common problem in society, and their prevalence and severity is increasing with age. Calibrating the observations made in stroke patients against otherwise healthy participants is therefore important as it will allow the consideration of these findings in the context of care pathways and public health initiatives on sleep more generally.

Sleep difficulties and daytime sleepiness are frequently experienced in those with stroke. For example, questionnaire- and actigraphy-based studies report chronic sleep difficulties in 34–67% of patients^4,5^, with 48% satisfying the criteria for insomnia^6^. 17–34% of patients with stroke further report disturbed daytime functioning due to excessive sleepiness^4,7,8^ and fatigue (30–69%)^9–13^. Daytime electroencephalographic (EEG) measures in chronic motor stroke further indicate greater prevalence of slower frequencies in resting state wake EEG^14^, a phenomenon often interpreted as a marker of sleep deprivation^15^. Together these findings suggest that sleep and daytime sleepiness represent a continued problem for those with stroke. Critically, the subjective experience of sleep problems does not always map onto objective physiological sleep parameters. For example, in the study by Herron^14^, the EEG findings would suggest greater daytime sleepiness, however, the objective sleepiness ratings were similar to those of controls.

The considerations summarized above highlight the need for the acquisition of PSG data and physiological measures of daytime sleep propensity in patients in the chronic stroke phase of stroke and age-matched controls using two gold standard methodologies, polysomnography and the Multiple Sleep Latency Test (MSLT).

In the present study 22 community dwelling patients with first ever stroke at least 12 months prior to the data collection and 22 sex and aged matched controls were tested in a protocol comprising two consecutive nights of PSG conducted in the controlled environment of a sleep laboratory, followed by the MSLT after night two. The study enrolled patients with right-hemispheric stroke and sustained motor deficits to ensure a homogeneous cohort and to keep potential confounding factors to a minimum. For practical and ethical reasons, the motor deficit was mild since patients had to be able to dress and eat independently, and be comfortable within the laboratory setting. Physiological sleep characteristics indicative of sleep continuity and architecture, and the spectral EEG composition, were extracted from the PSG recorded on the second night. In line with best practice standards in PSG research, the first night served as an adaptation night and its data were not analysed. Daytime sleepiness measures comprised sleep latency and wake efficiency, as well as vigilance performance. In addition to the physiological data obtained through PSG and MSLT, participants also completed a sleep diary and wore actiwatches for two weeks prior to the first night in the sleep laboratory, and completed a number psychological scales, including the Beck Depression Inventory (BDI) and the Pittsburgh Sleep Quality Index (PSQI; see methods for details). Importantly these measures were obtained from patients and control partipants living independently in the community so that potential sleep difficulties in those living with the consequences of stroke could be compared to those living in similar age-typical situations, without the additional physiological and psychological challenges arising from having had, and living with the consequences of, a stroke.

## Results

### Subjective Sleep

Significant differences in global PSQI scores suggested poorer sleep in patients than controls (6.9 ± 3.1 vs 5.1 ± 2.8, F_1,38_ = 3.68, p < 0.05 with ŋ_p_^2^ = 0.09). Thereby patients were more likely to be categorized as poor sleepers (14/19 vs 10/21) when using the ≥ 5 PSQI criterion (odds ratio: 3.08, p = 0.049). Patients also reported significantly poorer sleep efficiency than controls (80.1 ± 10.3% vs 89.9 ± 10.8%, F_1,31_ = 7.1, p < 0.01; ŋ_p_^2^ = 0.186) in their sleep diaries. However, diary reports for total sleep time (patients: 422.6 ± 47.7 mins; controls: 433.3 ± 63.4 mins), number of wake periods (patients: 0.87 ± 9; controls: 0.9 ± 0.7), and daytime sleep (patients: 5.68 ± 8.7 mins; controls: 4.9 ± 9.4 mins) were not different between the groups.

### PSG

No significant group differences were observed for the apnea hypopnea index (AHI: patients: 9.8 ± 7.0; controls: 7.6 ± 7.5, F_1,38_ = 0.94, p = 0.37, ŋ_p_^2^ = 0.024) and periodic limb movements (patients: 12.08 ± 14.78, controls: 8.04 ± 10.13, F_1,38_ = 0.94, p = 0.31, ŋ_p_^2^ = 0.026).

Sleep stage parameters are shown in Table 1. Patients had significantly longer sleep latencies (23.9 ± 17.5 vs 15.9 ± 9.0 minutes; F_1,38_ = 4.43, p = 0.035, ŋ_p_^2^ = 0.083) and poorer sleep efficiency (72.0 ± 11.9 vs 79.1 ± 9.0; F_1,38_ = 4.70, p = 0.018, ŋ_p_^2^ = 0.110). At the same time patients had significantly more WASO (102.7 ± 44.4 vs 72.1 ± 40.8 minutes, F_1,38_ = 5.15, p = 0.014, ŋ_p_^2^ = 0.119) and more % wake than controls (20.6 ± 9.2 vs 14.7 ± 7.7%; F_1,38_ = 4.82, p = 0.017, ŋ_p_^2^ = 0.113). Lesion size did thereby not correlate with these variables.

**Table 1:** mean and standard error of sleep stage characteristics; ^*^time in minutes.

Sleep parameters	Patients	Control	difference
^*^Sleep latency (SL; Time to fall asleep after lights off)	23.9 ± 4.01	15.9 ± 1.96	8.0
Sleep period time (SPT; sleep onset to awakening (incl. wake))	497 ± 13.64	475.4 ± 12.6	21.6
Total sleep time (*TST; Time spend asleep (i.e. SPT- wake))	394.3 ± 14.49	403.3 ± 10.13	−9.0
Sleep efficiency (SE; % asleep as a function of time in bed)	72.0 ± 2.67	79.1 ± 1.97	−7.1
Wake since sleep onset (WASO; Time spent awake after sleep initiation)	107.2 ± 10.18	72.1 ± 8.90	30.6
% N1; % stage 1 in SPT	15.4 ± 1.88	13.3 ± 1.98	2.1
% N2; % stage 2 in SPT	45.5 ± 2.54	49.4 ± 2.51	−3.9
% N3 (slow wave sleep; % stage 3 in SPT)	1.4 ± 0.46	4.0 ± 1.84	−2.6
% REM (rapid eye movements; REM sleep in SPT)	17.2 ± 1.01	18.3 ± 1.15	−1.1

The remaining variables showed a numerical pattern of group differences pointing towards poorer sleep in patients, however these numerical differences were not significant. The spectral analysis revealed significantly greater gamma power during NREM sleep in patients than contols (F_1,38_ = 3.52, p = 0.034, ŋ_p_^2^ = 0.085).

### MSLT

**Figure 1:**
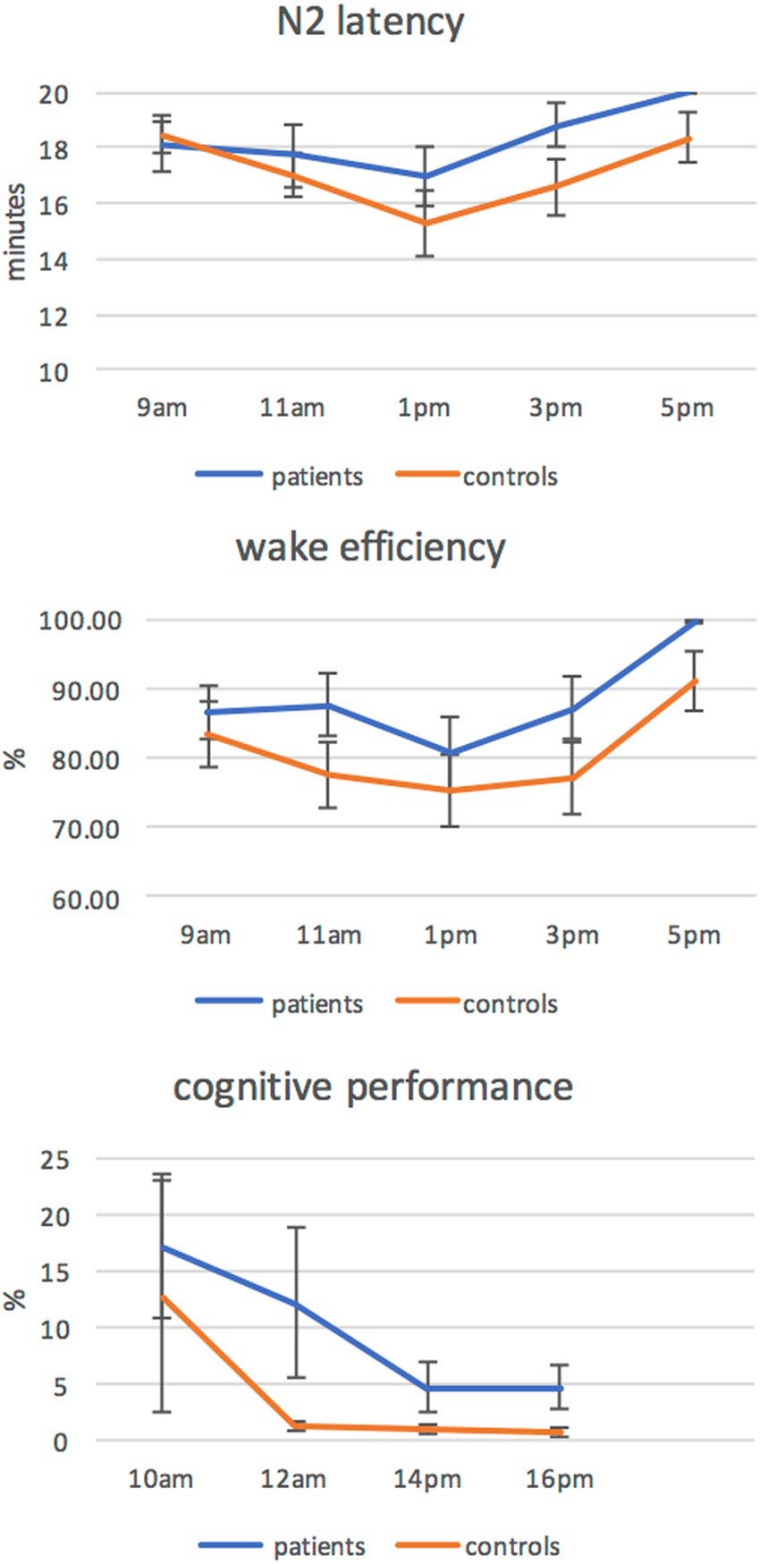
Top 2 panels shows makers of sleep propensity obtained from the MSLT for patients and controls. The bottom panel shows the error rates obtained in the TAP.

The data is illustrated in Fig. 1. Wake efficiency showed main effects for session (F_1.38_ = 5.95, p = 0.01, ŋ_p_^2^ 0.135) and group respectively (F_1.38_ = 4.91, p < 0.01, ŋ_p_^2^ 0.114); the session x group interaction was non-significant (p = 0.89). N2 latencies showed a significant session effect (F_3,114_ = 4.2, p < 0.01) but no group effect or interaction. However, visual inspection of the means suggested a tendency for longer N2 latencies in patients towards the end of the day. We therefore calculated the ANOVA across the timepoints 11am, 1 pm, 3 pm and 5 pm. This revealed a main effect of group (F_1,38_ = 3.9, p < 0.05) with significantly longer N2 latencies for patients at 1 pm, 3 pm, and 5 pm. Subjective sleepiness ratings (KSS) ranged from 2.2 to 3.7 for patients and 2.2 to 3.5 for controls and were not significantly different at group level.

The group x session ANOVA of the vigilance performance data (TAP) revealed a main effect session (F_1,38_ = 2.82; p = 0.021; ŋ_p_^2^ = 0.069) for errors, but no main effect group or interaction, and no effects for omissions. Subsidary analysis through planned t-tests further indicated similar errors in the two groups for the 10am session (t = 3.41, p = 0.37) but significantly poorer performance in patients for the noon (t = 1.76, p = 0.04), 2 pm (t = 1.82, p = 0.04) and 4 pm sessions (t = 2.13, p = 0.02). For omissions, patient performance was significantly poorer the 2 pm session only (t = 2.00, p = 0.026).

## Discussion

The present study examined physiological characteristics of sleep and daytime sleepiness in chronic stroke patients compared to age-matched healthy controls living in the community. Results from a 2-night PSG protocol followed by the Multiple Sleep Latency Test revealed that compared to controls, patients differ in measures of sleep continuity, sleep architecture, and spectral EEG characteristics, and are less likely to fall asleep during the day as indicated by the MSLT. In detail, the sleep EEG indicates longer sleep latencies and lower sleep efficiency. This was reflected by more time awake since sleep onset and greater % wake. At the same time, total sleep time was similar between the groups. Nocturnal EEG power was greater for patients in higher frequency bands. During the day patients fell asleep less easily but were more prone to errors in a vigilance task. These group differences were observed despite similar levels of sleep disordered breathing and periodic limb movements in both groups. In addition patients consistently reported poorer sleep efficiency in their sleep diaries and were more likely to be categorized as poor sleepers on the PSQI. However, subjective measures of fatigue and daytime sleepiness were similar between the two groups, highlighting a discrepancy between sleepiness perception and physiological sleepiness measures. Importantly, the present study found that sleep efficiency was reduced in patients while total sleep time was similar to controls. Total sleep time represents a purely physiological measure of sleep continuity, while sleep efficiency represents a superimposition of physiological and behavioral factors. This suggests that the lesions caused by the stroke have not impacted the synchronized slow oscillations in the cortico-thalamo-cortical feed-back loop relevant for effective sleep-wake regulation^16^, and hence renders the explanation of a lesion-induced ineffective sleep-wake regulation less likely. However, this interpretation is most likely only true for mild strokes, and further research is clearly necessary to ascertain the proposition that poorer sleep in chronic stroke may, at least in a subset of patients, occur without damage to sleep-wake regulating networks.

To the best of our knowledge the present study is the first PSG-MSLT study in community-dwelling patients with a chronicity >12 months. Our findings extend and substantiate the existing data for sustained sleep difficulties in chronic stroke by providing objective physiological evidence in a well-controlled sample. Taken together these findings suggests that normal sleep homeostasis, i.e. increasing daytime sleepiness after poor nighttime sleep, is compromised in patients. Reduced sleep continuity and increased power in higher frequency bands at night, combined with greater wake efficiency during the day, are typical characteristics of insomnia^17^. Our results may therefore be interpreted as reflecting a form of insomnia.

The group differences observed in the present study were obtained by contrasting a relatively healthy stroke population (i.e. relatively low levels of depression, no sleep-affecting drugs) with a group of healthy ‘normal sleepers’ rather than ‘good sleepers’. The latter is often used in sleep research with the rational that good sleep is the healthy state and should hence be used for benchmarking sleep in other groups. Here we chose not to go down this route to avoid an overestimation of group differences. The recruitment strategy for controls further had an element of recruitment bias such that those more conscious about sleep (e.g. because their sleep is not as good as they would like) were more likely to notice flyers and respond to them. While this argument also holds in principle for patients, the fact that they were actively contacted via the clinic and given information about the study outright makes a self-selection bias towards poor sleepers less likely for patients than controls. The group differences observed in the present study are therefore likely to be more pronounced in the wider population.

The present findings were obtained from patients living independent lives in the community rather than patients residing in rehabilitation or other care settings at the time of the experiment. This is an important aspect of our study because it makes the two study groups more comparable and hence less prone to group-specific confounding factors. Because both groups were of good psychological health, and both had low levels of depression, it is unlikely that the poor sleep observed in patients can simply be explained by differences in mental health status.

In the present study all patients had a right-hemispheric lesion. The sleep staging was therefore conducted for electrode C3 over the left (nonlesioned) hemisphere. While generally the non-lesioned hemisphere is considered to be ‘healthy’, the possibility of compensatory changes in homologous areas and/or the wider cortex is genuine, and these changes might have affected the EEG signal and hence the PSG parameters. However, the changes observed for the PSG parameters were not uniform but specifically affected certain aspects of sleep physiology. Moreover, the objective indicators for an insomnia-like disorder was corroborated by the sleep diaries and the ratings on the PSQI. We would therefore argue that any differences in the EEG signal caused by the lesion are unlikely to represent an epiphenomenon but are functionally relevant to sleep. This argument is underpinned by the absence of significant correlations between lesion size and sleep parameters.

One model frequently used to explain the pathomechanisms of insomnia is the hyperarousal model^18^. This model notes the importance of physiological and psychological factors in the disease and treatment mechanisms of insomnia, and provides a possible explanatory framework for the present findings. Specifically, we propose that the insult induces physiological and psychological changes that are prone to trigger disturbed sleep in the early phases of stroke recovery. These early sleep disturbances are well documented^3,19,20^. Moreover, depression and fatigue are highly prevalent in the acute and postactue phase^21,22^, and both aspects can negatively affect and/or interact with sleep^23–25^. Perpetuating factors arising from psychological processes, such as maladaptive behaviors and dysfunctional beliefs, as well as physiological aspects including lesion characteristics and medication, can maintain these early sleep difficulties and lead to a persistent sleep disorder in the chronic state as predicted by Spielman’s 3 P model^26^. Should these assumptions be correct it would follow that early psychological intervention could reduce the likelihood of sustained sleep difficulties in the chronic phase of stroke. The potential role for psychological or bhavioural factors in disease maintenance is supported further by our observation that sleep efficiency but not total sleep time is reduced in patients. Both measures are reflective of sleep continuity. However, while total sleep time represents a purely physiological measure, sleep efficiency represents a superimposition of physiology and bedtime behaviour.

An alternative explanation to the one offered above could be a general reduction of sleep pressure resulting from lower levels of physical activity in patients. This, however, is unlikely to fully explain the findings. Firstly, because sleep efficiency was poorer in patients than controls, their tendency to fall asleep during the day should be higher than in controls. But the contrary is the case: patients have poorer sleep efficiency at night and greater wake efficiency during the day. Secondly the patients were relatively well recovered and lived an active life, a notion corroborated by the subscores on the stroke impact scale. However, the actigraphy data, obtained in the fortnight prior to the experiment, revealed significantly lower activity counts for patients than controls (199 ± 94 vs 315 ± 146; p < 0.01). While one could argue that actigraphy data in a person with hemiparesis is a questionable proxy for the level of physical activity, it is a genuine possibility that patients were less physically active, and that this might have negatively impacted their sleep homoestasis. However, the laboratory situation controlled critical zeitgebers, sleep-wake pattern and, to some extent, physical activity. Reduced sleep pressure therefore offers a limited explanation for the present data. The issue, however, highlights the contribution of health behaviors to the pathomechanism of post stroke insomnia, and hence potential pathways for non-pharmacological intervention.

In addition to the arguments above it is further noteworthy that actigraphy counts in patients with hemiparesis are typically calibrated against the less-affected limb, and this was not done in the present study. Measures from the affected limb are likely to underrepresent movement, which further strengthens the argument that sedentary lifestyle alone is unlikely to be an exhaustive explanation for the poor sleep efficiency observed in our study.

A further possible contributor to poorer sleep efficiency could be greater pain or discomfort in patients. While this possibility cannot be ruled out entirely, it is an unlikely explanation given that patients in the present study had mild hemiparesis and were screened for excessive pain.

While the present study was well matched and shows no significant differences for any of the critical demographic variables, it is true that numerically the stroke group contains more men, is older and has higher BMI. All these factors are typically associated with more disrupted sleep. On statistical grounds there is no room to argue that the sleep-related group differences were due to any of these factors, as none of them were significant. However, the possibility that the combined effect of the numerical differences in these three parameters might have contributed to the sleep difference between the groups, although unlikely, cannot be entirely excluded.

The latest NICE guidelines for stroke rehabilitation issued by the National Health Service in the UK do not mention sleep^27^. This omission stands in contrast to the recognized importance of sleep for physical and mental health^28^. We therefore argue that sleep is a concern in stroke and hence should be given greater consideration in stroke care. For example, poor sleep increases risks, such as the likelihood for cognitive failures or falls^29–31^. These risks, and the impact thereof, are likely to be aggravated in older persons with a disability (i.e. in this case stroke). In addition, age-related comorbidities, such as hypertension, and sleep are strongly linked^28,32–34^. Sleep disordered breathing represents the biggest single risk factor for recurrent strokes^35^. 29% of stroke survivors suffer clinical levels of depression^36^, a condition strongly associated with poor sleep^37^. In addition, sleep is conducive to skill learning^38,39^, while motor learning is known to be reduced in insomnia^40,41^, and hence likely to mediate rehabilitation outcome^3,42–44^. A better understanding of sleep in patients long after the incident, and, more importantly the mechanisms leading to these sleep difficulties might therefore represent an opportunity to improve quality of life and long-term outcome.

## Conclusion

The present study provides evidence for an insomnia-like sleep disorder in the chronic phase of stroke. Given the importance of sleep for daytime function, psychological wellbeing and health, as well as the proposed role in neurorehabilitation, these findings highlight the need to encapsulate sleep in stroke care provision. The pathomechanisms proposed here may not only be relevant to stroke but also other chronic medical conditions.

## Methods

### Participants

21 patients completed the study but two were excluded from further analysis because of technical problems (i.e. 19 patients; 13 males, 9 females; aged 63.5 ± 8 years). All patients had mild left-sided upper-limb hemiparesis following right-hemispheric first-ever stroke 13–108 months prior to the study (mean chronicity 26.9 months). Recovery, assessed at the time of study, was good as indicated by a mean global recovery score of 80.3 out of 100 on the Stroke Impact Scale, SIS. Clinical details and selection criteria are listed in Table 2. All participants were non-smokers and did not consume more than 2 units of alcohol per day. Patients taking sleep-affecting medication were excluded from the study.

**Table 2:** Selection criteria, patient characteristics and demographics.

**Inclusion criteria**					
Paresis of left upper limb					
Unilateral right-hemispheric stroke					
Time post-stroke >12 months					
Premorbid right-hand dominant					
**Exclusion criteria**					
Inability to clearly communicate/understand instructions					
Mini Mental State exam ≤25					
Inability to feed, wash or dress independently					
Sleep disorder determined through sleep interview					
AHI or PLMS >15					
Neurological or psychiatric comorbidities					
Seizures 6 months before participation					
Hemineglect					
Shift work or jet lag 4 months before participation					
Regular consumption of recreational drugs					
Sleep-affecting medication 2 weeks before participation					
**Patient characteristics**	**Mean ± SD**	**Range**			
Time post-stroke (months)	26.9 ± 21.7	13/108			
Global SIS score	80.3 ± 12.4	50/100			
SIS Strength	65.8 ± 18.6	25.0/100.0			
SIS Memory	59.6 ± 17.6	14.3/85.7			
SIS Emotion	62.5 ± 23.7	22.2/100.0			
SIS Communication	74.4 ± 20.9	28.6/100.0			
SIS ADL	81.6 ± 20.3	40.0/100.0			
SIS Mobility	72.3 ± 23.4	33.3/100.0			
SIS Hand Function	63.7 ± 30.5	20.0/100.0			
SIS Social Participation	65.7 ± 23.7	18.8/100.0			
Lesion volume*, ml	26.9 ± 41.0	0.8/158			
	**Patients**	**Controls**	**F /Χ** ^**2**^	**p-value**	**η**_**p**_^2^
sex (m/f)	13/6	14/7	0.014	0.906	
family status (in relationship/ living alone)	18/1	16/5	2.69	0.101	
Age	63.5 ± 8.0	59.2 ± 8.6	2.57	0.117	0.063
BMI	26.9 ± 3.6	25.3 ± 4.3	1.62	0.211	0.041
years in education	10.1 ± 1.6	10.4 ± 1.7	0.37	0.546	0.010
PSQI	6.9 ± 3.1	5.1 ± 2.8	3.68	0.063	0.090
BDI	5.8 ± 5.2	3.4 ± 4.1	2.64	0.113	0.065
AHI	9.8 ± 7.0	7.6 ± 7.5	0.945	0.37	0.024
PLMS (SPT)	12.08 ± 14.78	8.04 ± 10.13	1.03	0.31	0.026

Abbreviations: SIS = Stroke Impact Scale; ADL = Activities of daily living; AHI = Apopnea-hyponea index; PLMS = Periodic limb movement syndrome; *imaging data of 17 patients were available.

Patient volunteers were recruited via a database of patients previously treated in the Depatment of Neurology at the University of Freiburg Medical Center. Those fulfilling the selection criteria (see Table 2) were invited by letter to contact the sleep laboratory. Interested candidates were telephone-screened before completing a full medical and psychological screening conducted by two of the authors (D.E. & S.F.).

**Figure 2:**
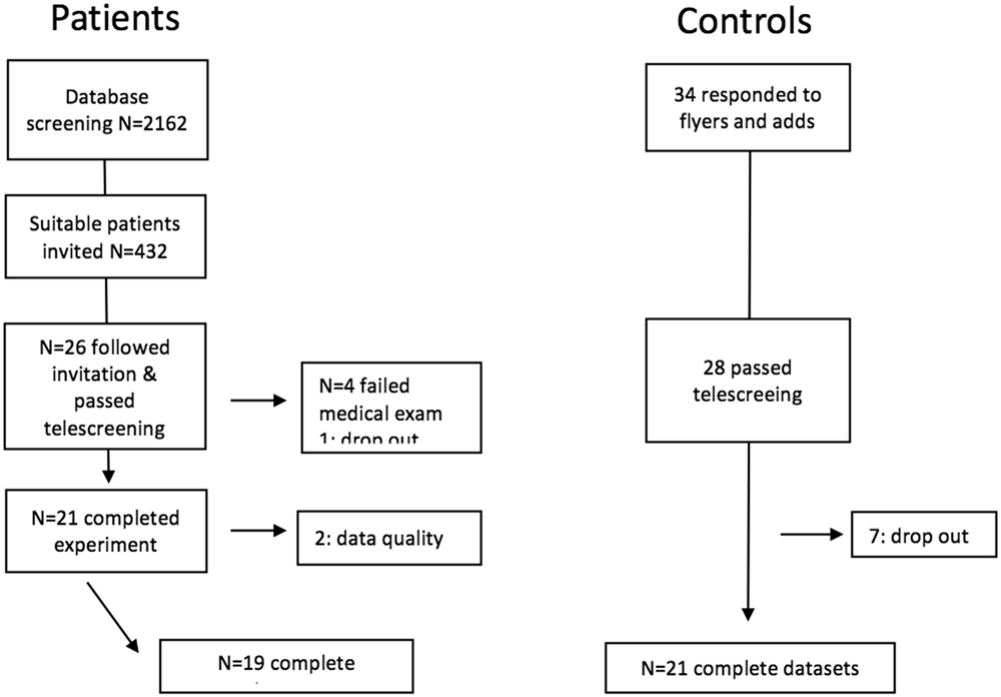
recruitment flow chart.

**Figure 3:**
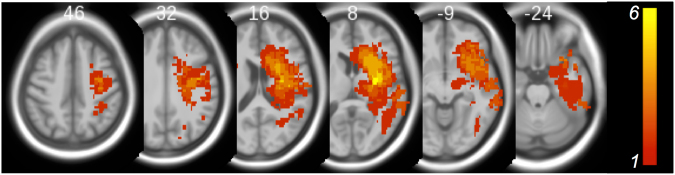
Lesion overlap obtained from diffusion-weighed images (1.5 T Siemens Avanto) acquired during routine diagnostics in the acute stroke phase. Using SPM8 (http://sourceforge.net/projects/spmtools/) lesions were delineated with MRIcron (http://www.cabiatl.com/mricro/mricron/index.html) and normalized into standard MNI-space (the Z-coordinates are provided). Color bar indicates the number of overlapping lesions.

The control group comprised 21 age (±5 years) - and sex-matched healthy volunteers, recruited through advertisements in local newspapers, flyers in public places, word of mouth and websites. The protocol for tele- and pre-screening, and the inclusion/exclusion criteria were similar to those for patients. Group statistics of demographic variables are provided in the lower part of Table 2. Participant enrollment and the study overview are presented in Fig. 2. Magnetic resonance imaging (MRI) recordings were obtained during routine diagnostics and used to extract lesion charateristics (Fig. 3).

The study was approved by the University of Freiburg Medical Center Ethics Committee, and conformed to the Declaration of Helsinki. Written informed consent was obtained prior to the study. Participants received financial reimbursement for participation.

### Scales and Questionnaires

The Beck Depression Inventory (BDI^45^), the Pittsburgh Sleep Quality Index (PSQI^46^) and the Motor Activity Log (MAL^47^) were administered at screening. Subjective sleepiness during the day was assessed using the Karolinska Sleepiness Scale^48^ (KSS). A regular sleep wake pattern was ensured through sleep diaries and actigraphy obtained for two weeks prior to the study^49^.

### Polysomnography and Multiple Sleep Latency Test (MSLT)

**Figure 4:**
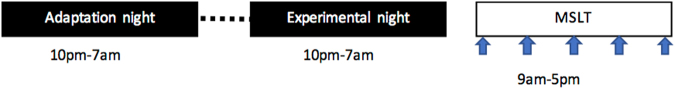
Flow chart of protocol. Participants arrived at the laboratory in the afternoon of the adaptation night for a full medical check-up and were given dinner at 7 pm. PSG wire-up started thereafter. The montage on the adaptation night included a nasal airflow sensor to determine OSA. This sensor was not used in the experimental night because it is often uncomfortable for participants. In the morning after the adaptation night, participants were given breakfast and asked to return to the laboratory for the experimental night for 6.30, were given dinner and wired up. In the morning after the experimental night, the PSG was removed. Participants were given breakfast before the EEG was wired up for the MSLT starting at 9 am.

The protocol is illustrated in Fig. 4. PSG was recorded during the two sleep laboratory nights to standardized procedures of the sleep laboratory in the Department of Psychiatry and Psychotherapy at the University of Freiburg Medical Center^50^, using a 24-channel video-polysomnography system (Leonardo Polygraph, Sagura Medical Equipment, Germany). The first night served as an adaptation night and was not analysed. The setup included the EEG channels Fz, Cz, Pz, C3, C4, P3, P4, FP1, FP2, F3, F4, T7, T8, A1 and A2 as reference, submental electromyogram (EMG), vertical and horizontal electrooculogram (EOG) and electrocardiogram (ECG).

The MSLT used a similar set up. The protocol comprised five 20-minute sessions (9am, 11am, 1 pm, 3 pm, 5 pm), where participants lay in bed in a dim room while the EEG was recorded. In-between these sessions, the Karolinska Sleepiness Scale and the sustained attention subtest of the Test of Attention Performance (TAP^51^) were acquired at 10am, 12 am, 2 pm and 4 pm.

### Off-line Analysis

PSG data were 0.5 and 70 Hz bandpass filtered and resampled at 200 Hz for offline analysis. Data collected between lights off in the evening and lights on in the morning were segmented into 30 second epochs and scored manually at the C3-A2 derivation by accredited sleep technicians using AASM criteria (http://www.aasmnet.org/practiceguidelines.aspx). Sleep parameters were calculated as described in^52^ and comprised measures of sleep continuity, sleep architecture, and REM characteristics as specified in Table 2. For the MSLT, N2 latency and wake efficiency, defined as the ratio between time awake and sleep opportunity (20 minutes respectively), were extracted for further analysis.

Spectral analysis was performed on 4-second windows with 0.49 Hz resolution similar to^52^. The average power, referenced to average reference, was calculated for delta (0.1–3.5 Hz), theta (3.5–8 Hz), alpha (8–12 Hz), sigma (12–24 Hz), beta 1 (16–24 Hz), beta 2 (24–32 Hz), gamma (32–48 Hz), and computed for all sleep stages combined, as well as separately for N2 and REM.

### Statistics

ANOVAs were calculated for group comparisons. T-tests and Chi square statistics were used for demographic variables as appropriate. Correlations examined the association of lesion volume and sleep. Unless otherwise stated, significance levels for group comparisons refer to one-sided testing to reflect the directionality of the hypothesis and to take account for the clear indication of poorer sleep in patients as reflected in questionnaire-based research.
