# Author Correction: Post-stroke insomnia in community-dwelling patients with chronic motor stroke: Physiological evidence and implications for stroke care

**DOI:** 10.1038/s41598-018-36813-2

**Published:** 2019-03-15

**Authors:** A. Sterr, M. Kuhn, C. Nissen, D. Ettine, S. Funk, B. Feige, R. Umarova, H. Urbach, C. Weiller, D. Riemann

**Affiliations:** 1 School of Psychology, University of Surrey, Guildford, UK; 2 Department of Psychiatry and Psychotherapy, Medical Center - University of Freiburg, Faculty of Medicine, University of Freiburg, Freiburg, Germany; 3 University Hospital of Psychiatry and Psychotherapy, Bern, Switzerland; 4 Sleep Wake Epilepsy Center, Neuroscience Center, Bern, Switzerland; 5 Department of Neurology, Medical Center, University of Freiburg, Freiburg, Germany; 6 Department of Neuroradiology, Medical Center - University of Freiburg, Faculty of Medicine, University of Freiburg, Freiburg, Germany; 7 Department of Neurology, University Hospital Bern, Bern, Switzerland

Correction to: *Scientific Reports* 10.1038/s41598-018-26630-y, published online 30 May 2018

The original version of this Article contained errors in the affiliation list. Affiliations 2 and 5 were incorrectly listed as ‘Department of Psychiatry and Psychotherapy, Medical Center, University of Freiburg, Faculty of Medicine, University of Freiburg, Freiburg, Germany’ and ‘Department of Neurology, University of Freiburg, Freiburg, Germany’ respectively.

In addition, R. Umarova was incorrectly affiliated with ‘University Hospital of Psychiatry and Psychotherapy, Bern, Switzerland’ and ‘Sleep Wake Epilepsy Center, Neuroscience Center, Bern, Switzerland’.

H. Urbach was incorrectly affiliated with ‘Department of Neurology, University of Freiburg, Freiburg, Germany’.

The correct author affiliations are listed below:

Affiliation 1:

School of Psychology, University of Surrey, Guildford, UK

A. Sterr

Affiliation 2:

Department of Psychiatry and Psychotherapy, Medical Center - University of Freiburg, Faculty of Medicine, University of Freiburg, Freiburg, Germany

M. Kuhn, C. Nissen, D. Ettine, S. Funk, R. Umarova, B. Feige & D. Riemann

Affiliation 3:

University Hospital of Psychiatry and Psychotherapy, Bern, Switzerland

C. Nissen

Affiliation 4:

Sleep Wake Epilepsy Center, Neuroscience Center, Bern, Switzerland

C. Nissen

Affiliation 5:

Department of Neurology, Medical Center, University of Freiburg, Freiburg, Germany

C. Weiller

Affiliation 6:

Department of Neuroradiology, Medical Center - University of Freiburg, Faculty of Medicine, University of Freiburg, Freiburg, Germany

H. Urbach

Affiliation 7:

Department of Neurology, University Hospital Bern, Bern, Switzerland

R. Umarova

These errors have now been corrected in the PDF and HTML versions of the Article.

